# Screening of Cyclodextrins in the Processing of Buserelin Dry Powders for Inhalation Prepared by Spray Freeze-Drying

**DOI:** 10.34172/apb.2023.086

**Published:** 2023-07-11

**Authors:** Mostafa Rostamnezhad, Katayoon Mireskandari, Mohammad Reza Rouini, Samira Ansari, Majid Darabi, Alireza Vatanara

**Affiliations:** ^1^Department of Pharmaceutics, Faculty of Pharmacy, Tehran University of Medical Sciences, Tehran, Iran.; ^2^CinnaGen Medical Biotechnology Research Center, Alborz University of Medical Sciences, Karaj, Iran.; ^3^CinnaGen Research and Production Co., Alborz, Iran.

**Keywords:** Spray freeze-drying, Buserelin, Raffinose, Cyclodextrins, Microparticles, Aerodynamic behavior

## Abstract

**Purpose:**

In this study, we prepared inhalable buserelin microparticles using the spray freeze-drying (SFD) method for pulmonary drug delivery. Raffinose as a cryoprotectant carrier was combined with two levels of five different cyclodextrins (CDs) and then processed by SFD.

**Methods:**

Dry powder diameters were evaluated by laser light scattering and morphology was determined by scanning electron microscopy (SEM). Differential scanning calorimetry (DSC) and X-ray diffraction (XRD) analysis were utilized for the determination of crystalline structures. The aerodynamic properties of the spray freeze-dried powders were evaluated by twin stage impinger (TSI) and the stability of prepared samples was assessed under normal and accelerated conditions.

**Results:**

The prepared powders were mostly porous spheres and the size of microparticles ranged from 9.08 to 13.53 μm, which are suitable as spray-freeze dried particles. All formulations showed amorphous structure confirmed by DSC and XRD. The aerosolization performance of the formulation containing buserelin, raffinose and 5% beta-cyclodextrin (β-CD), was the highest and its fine particle fraction (FPF) was 69.38%. The more circular and separated structures were observed in higher concentrations of CDs, which were compatible with FPFs. The highest stability was obtained in the formulation containing hydroxypropyl beta-cyclodextrin (HP-β-16. CD) 5%. On the contrary, sulfobutylether beta-cyclodextrin (SBE-β-CD) 5% bearing particles showed the least stability.

**Conclusion:**

By adjusting the type and ratio of CDs in the presence of raffinose, the prepared formulations could effectively enhance the aerosolization and stability of buserelin. Therefore, they can be proposed as a suitable career for lung drug delivery.

## Introduction

 Pulmonary delivery of medicines is a promising manner due to its non-invasive characteristic for both systemic and local effects in the treatment of a wide range of disorders such as cystic fibrosis, chronic obstructive pulmonary disease, asthma, diabetes, etc.^1,2^ High surface area and lack of the first-pass metabolism are outstanding properties that contribute to selection of the respiratory tract as a suitable site for drug delivery.^3,4^

 Extensive studies in the last decade have shown that for some reasons such as lower proteolytic enzymatic degradation, high absorption of macromolecules in the lungs, rapid onset of action, etc., delivery of peptides and proteins through the lungs can be very effective. But the acquisition of inhalable peptides and proteins with suitable aerodynamic properties is associated with challenges.^5^

 Buserelin is a peptide analog of hypothalamic gonadotropin-releasing hormone (GnRH) used to treat premature puberty, prostate cancer, uterine leiomyoma, endometriosis and ovulation stimulation.^6,7^ Comprehensive application of GnRH analogs has been a reason for developing various dosage forms, including injectable solutions, slow-release systems and nasal spray.^8^ Due to the invasive nature of injection and the variation in the bioavailability of the drug through the nasal route (2.5% to 6%), it seems to be useful to obtain a dosage form that solves the problems of available buserelin products in the market.^9^

 Dried forms of protein and peptide drugs provide more stable formulations than liquid ones.^10^ A wide range of dry powder inhalers (DPIs) have been proposed for respiratory drug delivery. DPIs should provide appropriate mass median aerodynamic diameter and reduce particle adhesion, leading to an increase in drug deposition in the lungs.^11^

**Figure 1:**
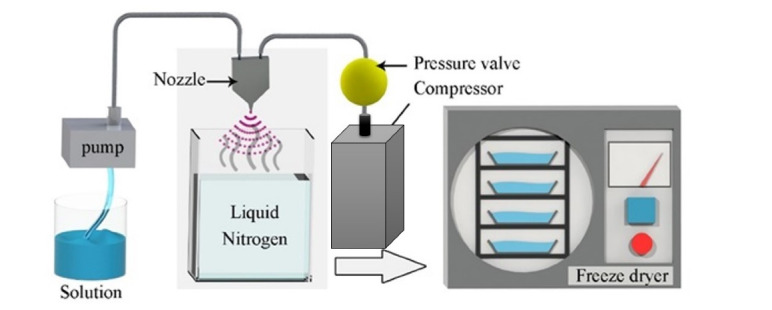
Schematic of SFD process

 There are a wide variety of particle engineering techniques that can be exploited for DPIs production, including micronization and blending, supercritical fluid processing, freeze drying, spray drying (SD) and spray freeze-drying (SFD).^12,13^ SFD is a relatively novel particle engineering technique, suitable for production of porous, fine and uniform particles. Therefore, it is an attractive method among different particle engineering methods.^14-16^ The general principle of this method is atomization of feed solution into liquid nitrogen and then lyophilization of frozen droplets to allow the solvent to be sublimated at low temperature and vacuum conditions (Figure 1).^7,8^ Spraying and rapid freezing of the droplets and sublimation of ice particles in this method create porous spherical particles. As a result, SFD method creates particles with suitable aerodynamic properties, size, density^17^ and leading to higher stability in the lungs and nasal mucosa than other conventional drying methods.^18^ This in turn improves performance of engineered drug delivery system. Also, the low applied temperature exploited in this method preserves the nature of drugs (especially protein and peptide drugs).^18,19^

 In contrast, there are some drawbacks to the SFD method. Several stresses are applied on the peptide and proteins during the SFD process can cause some irreparable damages on the molecule structures, causing aggregation and structural perturbations. Mechanical stress of spraying from the nozzle, the air-water interface formation and cold denaturation are some sources of damage in this process. To overcome this, different stabilizing agents are employed to prevent process instability^20-22^ and numerous studies have shown the protective effects of various sugars and polyhydroxy compounds on the stability of peptides and proteins during dehydration, freezing, and storage.^23-25^ As these excipients interact with applied drugs in different ways, further studies need to be implemented to introduce specific combinations for an individual drug and the aerodynamic behavior of each drug in the presence of different sugars is unique; So, studying various aspects of excipients can be helpful.

 The most common stabilizing agent utilized in dried formulations are sugars like lactose, mannitol, trehalose, raffinose and sucrose. Currently, raffinose has been used in some studies due to its desirable properties, more specifically, the formation of microparticles containing raffinose and a protein model, with proper aerodynamic and micrometric properties for pulmonary delivery.^26,27^ It provides several advantages over other common sugars such as trehalose and sucrose.^28^ Raffinose, is a non-reducing oligosaccharide preventing Millard’s reaction in proteins^26^ represents its merits via two mechanisms, water replacement and vitrification relating to the kinetic and thermodynamic aspects of stabilization, respectively.^29,30^ Two main parameters affecting the physical and chemical stability of biomolecules are glass transition temperature (Tg) and collapse temperature (Tc).^31^ Through water replacement mechanism, raffinose makes more effective direct hydrogen bonds with biomolecules than trehalose and sucrose.^23,32^ Finally, it is a suitable stabilizer for the long-term storage of dried formulations.^33-35^

 Cyclodextrins (CDs) as cyclic oligosaccharides are also appropriate excipients that can be employed to inhibit protein and peptide aggregation.^36-38^ While CDs at concentrations greater than 1% (w/v) act as lyoprotectant and stabilize protein by both water replacement and vitrification mechanisms, at concentrations of less than 1% (w/v) play as a non-ionic surfactant agent, protecting the protein and peptide from the air-water interface.^39^ It was found that CD-raffinose binary carriers can be exploited as an ideal candidate for inhaled drug delivery^27^ due to the improvement of anti-hygroscopicity and aerodynamic properties of dry powders prepared by SD method.

 Despite the fact that broad studies have been focused on CDs employment for particle engineering purposes, due to the structural diversity and different mechanisms of CDs to protect biologics at different concentrations, further investigations can provide valuable information about the impact of different CDs on physicochemical characteristics of fabricated particles. In this study, we aimed to evaluate the effect of combination of raffinose and five different CDs on the aerodynamic properties and stability of DPIs prepared by SFD process.

## Materials and Methods

###  Materials

 Buserelin was kindly donated by CinnaGen Company, Iran. Highly branch cyclic dextrin (HBCD) was obtained from Glico Nutrition Co. Ltd., Japan. Gamma-cyclodextrin (Gamma-CD) and sulfobutylether-β-cyclodextrin (SBE-β-CD), were purchased from CYCLOLAB 106. Ltd., Hungary. Beta-cyclodextrin (β-CD), Hydroxypropyl beta-cyclodextrin (HP-β-CD), raffinose, analytical grade of acetonitrile and phosphoric acid 85%, were purchased from Sigma, Germany. Size 2 hydroxypropyl methylcellulose (HPMC) capsules were purchased from Cipla, India.

###  Methods

####  Preparation of microparticles processed by SFD

 Firstly, aqueous solutions containing buserelin (4 mg), raffinose (400 mg) and five different CDs (HBCD, β-CD, HP-β-CD, Gamma-CD, and SBE-β-CD) with two ratios of CD: raffinose (0.05 and 5 % w/v) were prepared according to Table 1. In each experiment, 0.4 L of liquid nitrogen was poured into a 2 L glass container as cryogenic vapour. The feed solution was atomized into vapour above liquid nitrogen by a laboratory scale atomizer equipped with a pneumatic nozzle at a flow rate of 0.6 mL/min. After forming the frozen droplets at the bottom of the glass container and nitrogen evaporation, the lyophilization step was performed by a freezer dryer (Dorsatech, Iran). While primary drying was done at –50 °C and 0.005 mbar within 24 hours, the secondary drying took place in 24 hours and the temperature gradually reached to -20 °C. The lyophilized powders were gathered in glass vials for further evaluation.

**Table 1:** Types of feed solutions and particle size distribution for spray freeze-dried formulations

**Formulation**	**CD (%)**	**d**_10% _ **(μm)**	**d**_50% _ **(μm)**	**d**_90% _ **(μm)**	**Span**
F_1_	HBCD (0.05)	2.69	9.26	30.73	3.02
F_2_	HBCD (5)	2.61	9.08	29.74	2.98
F_3_	β-CD (0.05)	3.27	9.99	28.04	2.47
F_4_	β-CD (5)	2.38	11.65	29.81	2.35
F_5_	HP-β-CD (0.05)	3.43	10.75	30.38	2.50
F_6_	HP-β-CD (5)	2.91	13.53	30.91	2.06
F_7_	Gamma-CD (0.05)	1.79	11.76	30.38	2.43
F_8_	Gamma-CD (5)	1.74	11.22	28.96	2.42
F_9_	SBE-β-CD (0.05)	1.48	9.38	26.63	2.68
F_10_	SBE-β-CD (5)	2.01	11.04	27.93	2.34

####  HPLC analysis

 A reverse-phase high-performance liquid chromatography system (Knauer, Germany) equipped with a C_18_ column (4.6 × 150 mm, 5 µm particle size, Tosoh Bioscience, Japan) was used for buserelin assay. Analysis was performed under isocratic elution with mobile phase containing 75:25 (v/v) phosphate buffer/acetonitrile, pH 2.5. The detection wavelength, flow rate and injection volume were 220 nm, 1 mL/min and 50 µl, respectively. The LOD and LOQ of this method are 30 and 100 ng/mL, respectively. HPLC analysis is based on the buserelin monograph in the European Pharmacopoeia.^40^

####  Laser light scattering for particle size analysis

 For particle size analysis by laser light scattering instrument, (Sympatec GmbH,Germany), 10 mg of each spray freeze-dried formulation was dispersed in 5 mL of acetonitrile as a dispersion medium before they were sonicated in a sonicator bath (Starsonic, Italy) for 5 minutes. In three measurements, the average particle size was obtained at obscuration between 15% to 20%. The span, a volume-based size distribution parameter, was obtained by Eq. 1:


Eq. (1) $$Span=\frac{D0.9-D0.1}{D0.5}$$

####  Scanning electron microscopy (SEM)

 The morphological characteristics of spray freeze-dried particles were examined by SEM at an accelerated voltage of 25 kV. (XL30, the Netherlands). The processed powders were poured on aluminum stub covered with carbon tape, then sputter-coated with gold at room temperature under vacuum (BAL-TEC, Switzerland).

####  Differential scanning calorimetry (DSC)

 Thermal behaviour and crystallinity of SFD processed powders were assessed by DSC apparatus (Mettler Toledo, Switzerland). Approximately, 10 mg of each sample was placed in the aluminum pan, sealed and then heated from -20 to 200 °C. Scan speed was 10 °C per min.

####  X-ray diffraction (XRD) analysis

 X-ray diffractometer (SmartLab, Rigaku, Japan) was applied to affirm the DSC results on the crystallinity of the powders. Samples were evaluated with Cu Κα at 40 kV with 30 mA and were scanned from 5 to 100 °C. Scan speed was 5 °C per min.

####  Particle density

 The bulk density of the selected formulations was measured by determining the volume of a known mass of the sample that has been poured into a 10 mL measuring cylinder. The true density was also measured using a helium pycnometer (Multipycnometer, Quantachrome Instruments, USA) after calibration of the instrument using standard stainless steel spheres provided by the manufacturer. Each formulation was analyzed three times.

####  In vitro aerodynamic behavior

 To compare the *in vitro *aerodynamic behavior of all samples, the twin stage impinger (TSI) was used. Then, for a more detailed investigation, three selected formulations were evaluated by Andersen cascade impactor (ACI).

 Approximately, 5 mg of each powder which contains 50 µg of buserelin was poured into an HPMC capsule and placed in a Cyclohaler^®^ device. The aerodynamic properties of the SFD processed powders were evaluated by TSI at vacuum strength of 60 L/min for 5 seconds calibrated with a flowmeter (Erweka, Germany) according to the British Pharmacopoeia 2022.^41^ The capsule, device and various parts of the TSI were washed by mobile phase. The drug contents of each part were determined by RP-HPLC and then, the emitted dose (ED), fine particle dose (FPD) and fine particle fraction (FPF) for each formulation were calculated. ED is the amount of drug taken out of the capsule and device into TSI, the FPD is the amount of drug collected in stage 2 and the FPF is the FPD divided by ED multiplied by 100.

 For further clarification, the aerodynamic properties of the selected powders were evaluated by ACI (Copley Scientific, Nottingham, UK) at vacuum strength of 60 L/min for 4 seconds calibrated with a flowmeter (Erweka, Germany) at a pressure drop of 4 kPa. Approximately, 10 mL mobile phase was applied to the preseparator (conforming to USP apparatus 3) before analysis to avoid particle bouncing. The capsule, device and various parts of the ACI were washed by mobile phase. The drug contents of each part were determined by RP-HPLC and then, the ED and FPF for three selected formulations, were calculated. ED is the amount of drug taken out of the capsule and device into ACI, the FPF is defined as the percent fraction of ED with particle aerodynamic diameter < 3.2 mm; i.e. ED deposited on stage 2 to F.^42^

####  Stability studies

 For stability studies, the SFD processed samples were placed in sealed glass vials with parafilmand stored at 25 167. °C, 40% and 45 °C, 60% relative humidity. Drug recovery for each formulation was evaluated by RP-HPLC at 1 month and 3 months storage.

## Results

 The ultra-fast freezing step, different rheological and energy-mass transfer phenomena in SFD method lead to obtaining products with different properties from those of spray drying (SD) or freeze drying.^43^ The quality and characteristics of spray freeze-dried particles have shown a significant correlation with the type and ratio of formulation components.^44^

###  Particle size distribution evaluation

 The particle size distribution parameters of the prepared particles are presented in Table 1. Mean particle size of the formulations was between 9.08 to 13.53 μm. Due to the formation of porous particles in the SFD process, this particle size range can be acceptable for inhalation. Formulation F_6_, containing HP-β-CD 5%, showed the largest mean particle size with the lowest span (2.06) and formulation F_1_, containing HBCD 0.05%, had the highest Span (3.02).

###  Scanning electron microscopy 

**Figure 2:**
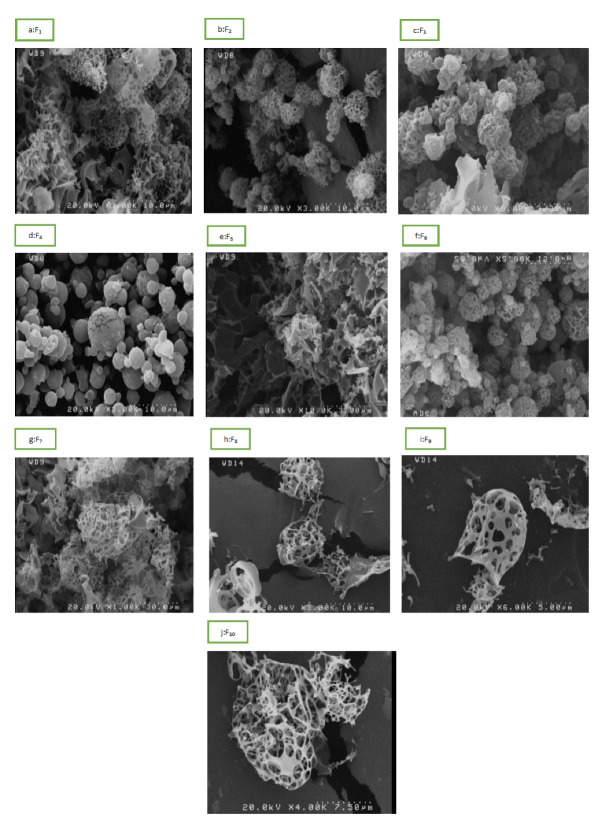
SEM images of spray freeze-dried formulations (a:F_1_; b:F_2_; c:F_3_; d:F_4_; e:F_5_; f:F_6_; g:F_7_; h:F_8_; i:F_9_; j:F_10_)

 The morphologies of microparticles fabricated by SFD technique were evaluated by SEM. According to micrographs (Figure 2), all formulations were formed in relatively spherical shape and pores within and on the surface. This fine porous structure is one of the most vital points of the SFD method compared to the conventional methods for particle engineering.^45^ Of course, the particles are different in terms of adhesion and porosity and there is a morphology difference as the concentration of components is changed. Except in formulations containing SBE-β-CD, in which the particles tend to be more aggregated and showed lower FPF, in other formulations, aggregation between particles decreased as the CD content increased from 0.05 to 5%. Also broken particles were found in the formulation containing SBE-β-CD 0.05%.

###  Differential scanning calorimetry 

**Figure 3:**
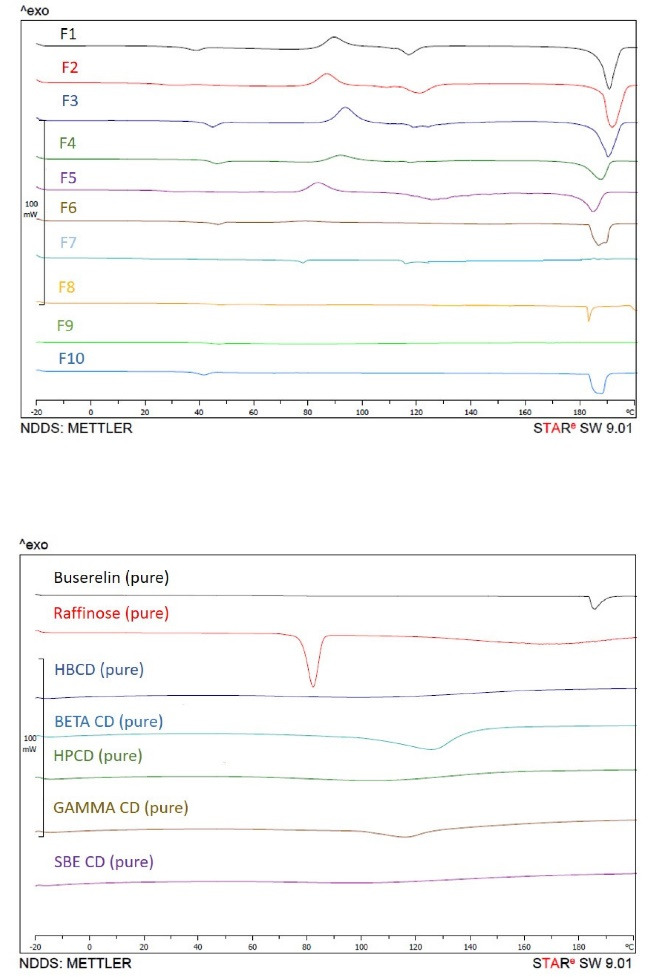
DSC thermograms of the spray freeze-dried formulations (a), and excipients (b)

 Thermal behavior of processed powders was assessed by DSC experiments. Figure 3 indicates the DSC thermograms of pure buserelin, pure excipients and processed powders with the combinations of raffinose and different cryoprotectants, including HBCD, β-CD, HP-β-CD, Gamma-CD and SBE-β-CD. Buserelin showed an endothermic peak around 180-190 °C, which was referred to its melting. This peak sharpness was seen in most formulations which could mean the buserelin remained crystalline after SFD. Moreover, there was a broad peak in some CDs between 100-140 °C, corresponding to water loss. Similarly, a sharp peak between 80-90 °C was seen for pentahydrated raffinose. This peak was not seen in the formulations since the raffinose became amorphous.

###  X-ray diffraction analysis

**Figure 4:**
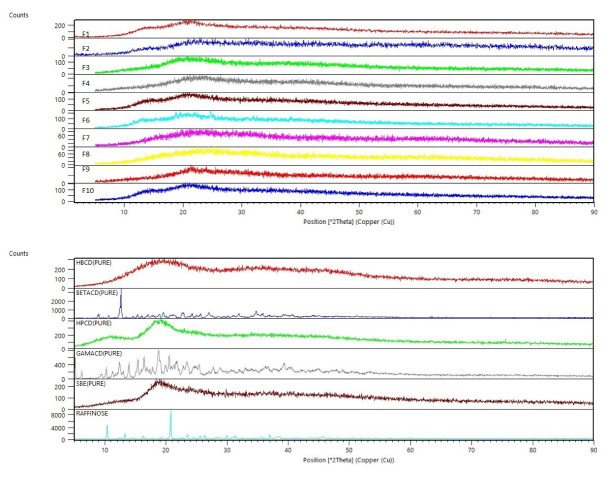
XRD patterns of spray freeze-dried formulations (a), and excipients (b)

 For confirmation of the obtained data from DSC, analysis by XRD was performed. As shown in Figure 4a, all spray freeze-dried formulations showed an amorphous structure; Whereas, pure excipients, raffinose and β-CD were crystalline (Figure 4b). Due to the rapid freezing of atomized droplets in contact with liquid nitrogen and rapid formation of particles, there is not enough time for crystallization and the amorphous habit of the particles is reasonable. Particle aggregation observed in SEM images is consistent with the amorphous nature of the particles.

###  Particle density

 The bulk density, true density, and tapped density values for selected samples were ranging from 0.081 to 0.095 g/cm^3^, 0.52 to 0.76 g/cm^3^, and 0.17 to 0.2 g/cm^3^ respectively (Table 2). Formulation F_4_ showed the lowest density values which was consistent with its highest FPF.

**Table 2:** Bulk density, true density and tapped density values of the spray freeze-dried samples.

**Formulation**	**Bulk density ** **(g/cm**^3^**)**	**True density ** **(g/cm**^3^**)**	**Tapped density** **(g/cm**^3^**)**
F_4_	0.081 ± 0.011	0.52 ± 0.002	0.17 ± 0.022
F_6_	0.092 ± 0.003	0.64 ± 0.021	0.18 ± 0.03
F_8_	0.095 ± 0.015	0.76 ± 0.017	0.2 ± 0.026

###  Aerodynamic performance of prepared powders

**Figure 5:**
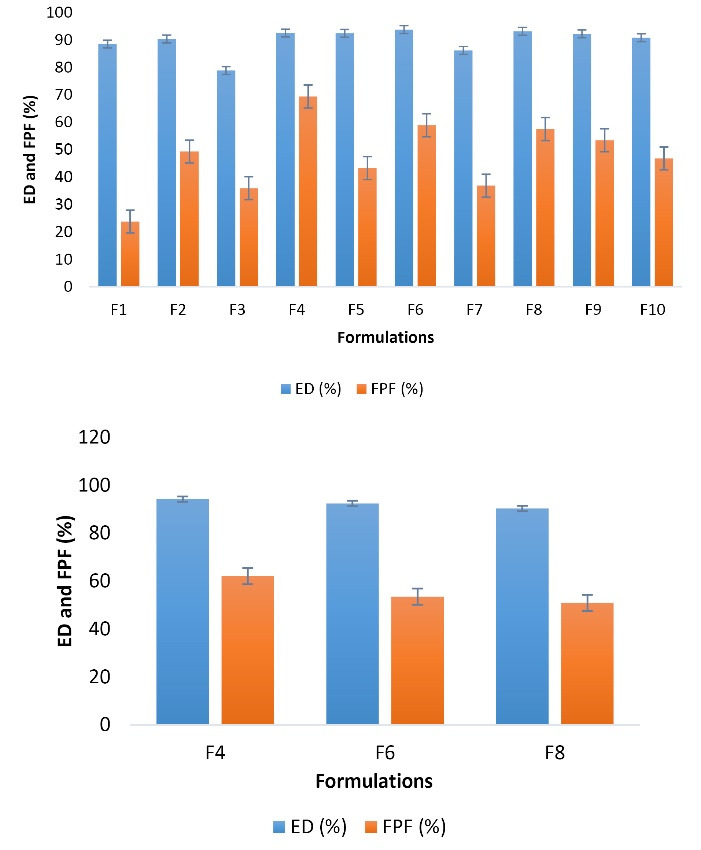
*In vitro* drug deposition results for spray freeze-dried formulations by TSI (a), and ACI (b)

 The *in vitro* aerosol performance of spray freeze-dried microparticles is illustrated in Figure 5. This study showed that the aerosolization of microparticles containing HBCD 0.05% had the lowest ED and FPF, 88.53% and 23.79%, respectively, while the FPF increased to 2-times for HBCD 5% (FPF = 49.32%). In contrast, β-CD 5% had the highest aerosolization property by far, with ED 92.53% and FPF 69.38%. The FPF value for gamma-CD 0.05% approximately halved (36.9%) and decreased to 1.2-times for gamma-CD 5% (57.52%). Although employing higher concentrations (5%) increased FPF amounts 1.3 and 1.08-fold for HP-β-CD and β-CD than 0.05%, respectively, it fell 12% for SBE-β-CD 5% compared to lower concentrations.

**Figure 6:**
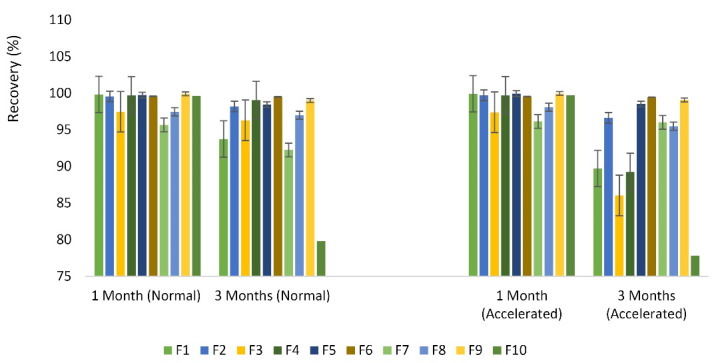
The recovery percent of spray freeze-dried formulations after 1 month, and 3 months of storage at the normal, and accelerated conditions (n = 3, Mean ± SD)

 The *in vitro* aerosolization behavior of selected formulations was determined using an ACI. ED% and FPF % data obtained for buserelin after aerosolization of spray freeze-dried formulations by using a Cyclohaler^®^ are shown in Figure 6. The results obtained by ACI were comparable with the results obtained by TSI. ED % of all three samples measured by this method was higher than 90%. The highest FPF value was related to β-CD 5% containing formulation (FPF = 62.1%). On the other hand, the β-CD 5% containing particles dispersed well, with most powders depositing at the lower stages of the ACI and having higher FPF compared to the two formulations containing HP-β-CD 5% and Gamma-CD 5%.

###  Stability evaluation

 The recovery percent of buserelin in the processed microparticles was assessed in normal and accelerated conditions for 1 and 3 months. The slightest changes were obtained for HP-β-CD 5% that reached to 99.58 and 99.50% for normal groups, 99.55 and 99.45% for accelerated samples in the first and third months. The most remarkable changes were related to SBE-β-237. CD 5%, reaching to 79.82 and 70% for normal and accelerated samples in the third month, respectively (Figure 6).

## Discussion

 Today, peptide and protein drugs are an important part of the pharmaceutical market. A large number of peptides and proteins are undergoing pre-clinical and clinical phases to enter the pharmaceutical market. However, most of these drugs are injectable, reducing the acceptance of these drugs, especially in the case of chronic diseases requiring long-term usage of drugs. To address this issue, inhalation drugs can be considered a desirable alternative to the injection route of administration due to the special characteristics of the respiratory system like very high surface area of the lung, low thickness of the alveolar membrane, and extensive blood supply to the lung tissue.^46^

 However, there can be found some challenges for inhalation drug delivery, including maintaining the chemical and structural stability of peptides and proteins during formulation and storage, preparing particles with desirable physicochemical properties, providing a deep lung penetration, and passing macromolecules through the epithelial membrane and entering the bloodstream.^47^

 The possibility of preparing particles for inhalation employing the SFD process was demonstrated in 1998 by Maa et al. Despite the unique ability of this method to prepare DPIs containing peptides and proteins (due to lack of exposure to high temperatures), few studies have been conducted in this field.^48^

 As the interaction of each drug with excipients (e.g., sugars and CDs) is unique, their effects on the aerodynamic properties and stability of the drugs will be different. Therefore, it is worthwhile to study different aspects of these excipients in the SFD process. This study is focused on the processing of formulations containing buserelin, fabricated by the SFD method for pulmonary drug delivery applications. To stabilize the denaturation of biomolecules through the SFD process, some stabilizing compounds have been used. The notable ability of sugars in protecting peptides and proteins has been proven in several studies.^13,49^ CDs are. other additives that can be effective in stabilizing peptides and proteins. We hypothesize that the combination of these two stabilizing agents (raffinose and CD) can provide a more stable formulation for buserelin with improved aerodynamic behavior against stresses applied to proteins and peptides during the SFD process.^27^

 In this study, five types of CDs (β-CD, HP-β-CD, HBCD, Gamma-CD, SBE-β-CD) and raffinose are employed to improve and optimize the aerodynamic properties and stability of microparticles containing buserelin fabricated by the SFD method.

 According to our results, as the CD concentration increased the FPF, ED and aerodynamic performance improved (except SBE-β-CD). These results can be referred to as the presence of more CDs on the surface of the frozen sprayed droplets and lower moisture absorption. The effects of CDs on the properties of proteins and peptides are variable due to different structures and the presence of other hydrophobic amino acids on their surface, so the effects of CDs on various proteins and peptides should be assessed.

 In all formulations, the FPF values were above 23%. According to the results, it can be concluded that all prepared formulations had relatively acceptable inhalation properties. The best formulation in terms of inhalation properties was related to the F_4_ formulation, containing β-CD 5%, with FPF = 69.38% (by TSI). The results obtained by ACI had the same trend as the results obtained by TSI. The β-CD 5% containing formulation with the most powders depositing at the lower stages of the ACI had higher FPF compared to the formulation containing HP-β-CD 5%. Several studies have shown that high concentrations of HP-β-CD reduce the melting point of proteins and may impair their aerodynamic properties.^50,51^ Also, HP-β-CD molecules have hydrophobic chains, deposited on the surface of particles. This can lead to powder adhesion and reduce FPF in the presence of hydrophobic interactions. The adhesion of microparticles of the F_6_ formulation can be seen in their SEM images and the increase in HP-β-CD resulted in a decrease in FPF compared to the F_4_ formulation (our optimal sample).

 The average ED in all selected formulations is above 78%. The obtained high amount of ED indicates a low level of electrostatic attraction and the efficiency of the SFD method in reducing this force. Moreover, this high ED is a sign of the low moisture content of the powder. As moisture is absorbed, the capillary forces prevent the powder particles from opening and leaving the capsule correctly.^52^

 The used combination of CD and raffinose can be effective in preventing the absorption of moisture. The combination of these two excipients could create a core-shell complex in which the raffinose is placed in the core part of this structure. In other words, a micelle-like structure can be created in which raffinose molecules are enclosed by CD molecules. According to this theory, CDs that are more hydrophobic than raffinose are more prone to move to the outer surfaces of atomized droplets and form the core-shell structure. Finally, with the drying of atomized droplets, CDs accumulate on the surface of the dried particles, resulting in limited molecular mobility and lower moisture access to raffinose in this micelle-like structure. That is an effective way to reduce moisture absorption and improve the aerodynamic behavior of microparticles.^27^

Equation 2 shows the calculation of aerodynamic diameter with respect to geometric diameter.


Eq. (2) $$d_{ae}=d_{p}{\left(\rho_{p}/\rho_{0}\chi \right)}^{1/2}$$

where d_ae_, d_p_, ρ_p_, and ρ_0_ are aerodynamic diameter, geometric diameter, particle density, and reference density (water density ~ 1000 mg/cm^3^), respectively. The shape factor, χ, depends on the sphericity of the particles. This factor is equal to 1 for spherical particles and higher than 1 for non-spherical particles.^53^

 SEM images show that the prepared particles are very porous and therefore ρ_p_ is very small. As a result, the aerodynamic diameter is significantly smaller than the geometric diameter. It has been proven that if the particles are spherical, depending on the porosity and density, the aerodynamic diameter, is about 20%-40% of the geometric diameter.^44^ So, the inhalation properties of DPIs are not only dependent on the particle size but also the shape and porosity play an important role. According to the mentioned cases, despite the relatively large apparent diameter (9 μm to 13 μm), the fabricated particles showed low aerodynamic diameter and suitable inhalation properties.

 In a study on terbutaline and salbutamol, it was shown that despite the geometric diameter of 41 μm of SFD-prepared particles, a significant number of particles had proper aerodynamic diameters to penetrate into the lungs. In fact, the lower surface-to-volume ratio of particles in the SFD method results in a less adhesion force between the particles and thus facilitates particle dispersion.^54^

 Maa et al presented a comparison between the aerodynamic properties of rhDNase and anti-IgE particles prepared by SD and SFD methods. Under the same atomization conditions, it was shown that the FPF values of spray freeze-dried particles (10 μm, porous particles) was significantly higher than that of spray dried particles (3 μm, dense particles). The FPF values reached 46% in spray dried particles to about 70% in spray freeze-dried particles. These observations can prove the theory that porous particles have a lower aerodynamic size and therefore exhibit more suitable inhalation properties.^55^ During SD, water leaks out of the droplets, causing the particles to shrink, reducing the original geometric diameter. In contrast, the particle diameter increases slowly during freezing in the SFD method. As a result, according to equation 1, it is evident that if particles with the same solid content are processed by both SD and SFD processes, the particles prepared by SFD have a lower aerodynamic diameter.^44^

Figure 2 shows the morphology of particles containing buserelin. According to the intact structure of the particles and lack of damage during the collection and preparation of samples, it can be said that these particles have good structural strength. There are many studies on the surface and internal structures of spray dried powders. In contrast, fewer studies have been performed on the microscopic structure of spray freeze-dried particles as it is a newer manner of particles fabrication. As shown in Figure 2, almost all samples have a very porous structure. Despite the large size of the particles, their highly porous structure with low densities makes them ideal for inhalation. Furthermore, the large size of these particles helps them escape from the alveolar macrophage system.^56^ During SFD, the dispersed droplets are frozen rapidly, resulting in the formation of ice crystals. Sublimation of these ice crystals leads to the formation of porous particles. In SEM images, some formulations represent adhesion or fusion of particles due to the amorphous structure of the processed microparticles. The existence of these amorphous structures has been confirmed by DSC and XRD analysis.

 The amorphous phase of microparticles can be suitable for maintaining the structure of peptides and proteins.^57^ According to the thermal evaluation of SFD-derived microparticles, an amorphous structure is obtained in the presence of CDs. The exotherm at about 100 °C can be due to the crystallization or rearrangement of raffinose molecules, and the subsequent endotherm may be due to their melting (in DSC thermograms). Also, the ultra-fast solidification step in the SFD method reduces the possibility of crystalline structure, leading to the expansion of amorphous glass.^38^ In our study, this was confirmed by XRD analysis of spray freeze-dried powders.

 Chemical and physical degradation of peptides and proteins and their instability are major challenges in formulation, production, and storage. To maintain the biological activity of peptides and proteins, saving their chemical and structural stability is essential.^47^ Peptides and proteins are subjected to various stresses during drying and engineering processes that may lead to changes in their structure.^58^ Structural changes may lead to complete or partial destruction of the biological activity of the peptides or proteins. As a result, a lower dose of the drug reaches the patient. Also, degradation of peptides may lead to toxic or immunogenic compounds.^59^ During SFD, the peptide faces stresses such as exposure to the liquid-air surface, atomization, etc., which may affect its stability; Therefore, the use of stabilizers in the formulation seems necessary.^60^

 Sugars have been shown to have a significant effect on the stability of peptides and proteins during the lyophilization process. In fact, preserving the structure of peptides and proteins in aqueous solutions is the result of their interaction with water molecules through the formation of hydrogen bonds. In general, sugars can form hydrogen bonds with peptides and proteins and prevent their degradation during the drying process.^61^ CDs are other additives that can be effective in stabilizing peptides and proteins. Also, they are more likely to improve the bioavailability of drugs,^62^ leading to improve pulmonary drug delivery efficiency. Biocompatibility of CDs has been proven in many studies in which there was no significant tissue damage.^63^

 Raffinose is more effective in forming hydrogen bonds with biomolecules than compounds such as trehalose and sucrose. The collapse temperature (Tc) of formulations containing raffinose is higher than sugars such as maltose, lactose, trehalose, and sucrose. Also, it increases the Tg in the remaining amorphous matrix. Due to the thermophysical properties of raffinose, this material is preferred as an excipient and stabilizer in particle engineering processes.^26^

 Moreover, the concentration of HP-β-CD has a significant effect on the stability of buserelin. The recovery percentage of buserelin from processed powders increases with increasing HP-β-CD ratio in the formulation. Numerous studies have been conducted on the stabilizing effects of various types of CDs on peptides or proteins during SD and lyophilization.

 CDs in lyophilized formulations have been used in very different concentrations from 0.0001% (w/v) to 10% (w/v). Based on different concentrations of CDs employed in lyophilized formulations, they can be a cryoprotectant or a surfactants. At high concentrations, CDs play the role of lyoprotectant. Sugars are stabilizing agents through the mechanism of “water molecule replacement”. If the purpose of using CDs is their lyoprotectant role, they should be used at least in a weight ratio equal to peptide or protein.^28^ Serno et al showed that at low concentrations (less than 1%) the surfactant effect of CDs is more pronounced.^64^

 The surfactant effect is used to maintain the structure of peptides and proteins during atomization and drying. Surfactants reduce the contact of proteins and peptides with hydrophobic surfaces (e.g, the surface between water-air or water-ice) and prevent their structural disruption and accumulation.^65^ The pattern obtained in the stabilization of peptides and proteins seems to be true during the lyophilization process in SFD. In this regard, similar studies have been performed by this team on the effect of CDs on the stability of IgG, calcitonin, and parathormone during SFD. The results represented the significant effects of CDs on improving the stability of these drugs compared to CD-free formulations.^22,49,66^

 Determining the exact ratio of CD: peptide or protein depends on the type of peptide or protein as well as other components of the formulations. Among different types of CDs, β-CD, HP-β-CD, Gamma-CD, SBE-β-CD, have shown the highest level of safety in injectable compounds.^67^ Studies have shown that the ability of CDs to stabilize peptides and proteins is varying. β-CD derivatives are more efficient at stabilizing peptides and proteins than alpha and gamma CDs, which may be due to the greater compatibility of amino acid benzene groups with the internal cavity of β-CD derivatives.^38^

 The addition of SBE-β-CD to the formulations (formulations F_9_ and F_10_) resulted in less stability than other formulations. It has been shown that the ionic strength of CDs has a significant effect on the stability of peptides and proteins. Due to the higher negative charge in SBE-β-CD, the migration of these CDs to the surface of microparticles in the core-shell structure decreases, which can be lead to lower stability and poor aerodynamic performance of microparticles.^68^

 The best stability results of this study are related to the F_6_ formulation. The protection of the peptide can be due to the hydroxyl groups in HP-β-CD and the formation of hydrogen bonds. HP-β-CD with a concentration of 5% with higher properties of “replacement of water molecules”, offers better stability compared to the formula F_5_. In our previous study, a combination of trehalose and HP-β-CD was used to prepare DPIs containing calcitonin by the SFD technique. HP-β-CD significantly improved the stability of calcitonin, which may be due to the surfactant effect of this CD.^50^ The formation of protein or peptide complexes by HP-β-CD is greater than β-CD because more hydrophobic segments are formed within the complex and provide deeper cavities due to the hydroxypropyl groups. Also, the surfactant property of HP-β-CD is higher than β-CD, so it has more competition for placement at air-water interfaces and provides better protection.^69^ Also, in a study on the stabilizing effect of various CDs on lactate dehydrogenase during the lyophilization process, it was found that HP-β-CD had the greatest stabilizing effect.^70^ The results show that branched CDs such as HP-β-CDs can stabilize peptides or proteins better than β-CD. Comparing the results of this study with other studies, it was found that the effect of CDs on aerodynamic properties and stability of microparticles varies according to the type of peptide. Due to the mechanism of action, CDs were used in two ratios of 0.05 % and 5% with raffinose and at higher CD percentages, the aerodynamic properties and stability of the formulations were improved.

## Conclusion

 In this study, inhalable microparticle containing buserelin, raffinose and five different CDs (HBCD, β-CD, HP- β-CD, Gamma-CD, SBE- β-CD) were fabricated via SFD method (F_1_-F_10_). The morphology of prepared microparticles was semi-spherical with a rough, porous surface. The highest stability of microparticles was obtained for HP-β-CD 5% in normal and accelerated conditions after one and three months. The highest aerosolization property was showed for β-CD 5%. Overall, the type and concentration of utilized CDs effectively impacted FPF and ED of produced microparticles. Generally, employing a higher concentration of CDs improved the FPF of microparticles. But the increase in HP-β-CD portions led to a decrease in FPF than sample containing β-CD 5% due to high concentrations of HP-β-CD may impair aerodynamic properties. As shown in previous studies, the amorphous matrix showed better protection for the proteins and peptides structures. The amorphous structure of all fabricated microparticles was confirmed by DSC and XRD evaluation. In conclusion, the employed SFD process and co-cryoprotectants can be utilized to prepare microparticles containing peptides like buserelin for lung delivery applications.

## Acknowledgments

 We wish to acknowledge Tehran University of Medical Sciences (TUMS).

## Competing Interests

 The authors report no declarations of interest.
